# The Tumor Microenvironment—A Metabolic Obstacle to NK Cells’ Activity

**DOI:** 10.3390/cancers12123542

**Published:** 2020-11-27

**Authors:** Joanna Domagala, Mieszko Lachota, Marta Klopotowska, Agnieszka Graczyk-Jarzynka, Antoni Domagala, Andriy Zhylko, Karolina Soroczynska, Magdalena Winiarska

**Affiliations:** 1 Department of Immunology, Medical University of Warsaw, 02-097 Warsaw, Poland; joanna.stachura@wum.edu.pl (J.D.); agraczyk@wum.edu.pl (A.G.-J.); andriy.zhylko@wum.edu.pl (A.Z.); karolina.soroczynska@wum.edu.pl (K.S.); 2 Postgraduate School of Molecular Medicine, Medical University of Warsaw, 02-091 Warsaw, Poland; 3 Department of Clinical Immunology, Medical University of Warsaw, 02-006 Warsaw, Poland; mieszko.lachota@wum.edu.pl (M.L.); marta.klopotowska@wum.edu.pl (M.K.); 4 Institute of Medical Sciences, Collegium Medicum, Jan Kochanowski University of Kielce, 25-317 Kielce, Poland; antoni.domagala@onkol.kielce.pl; 5 Department of Urology, Holy Cross Cancer Center, 25-734 Kielce, Poland

**Keywords:** NK cell, tumor microenvironment, metabolism

## Abstract

**Simple Summary:**

Natural killer cells are able to effectively eliminate tumor cells without previous sensitization, therefore the interest in their application in tumor immunotherapy has recently increased. However, tumor cells synergize with tumor-associated cells to create a specific immunosuppressive niche, which limits the activity of NK cells. In this review, we provide a detailed description of molecular mechanisms responsible for NK cells’ cytotoxic machinery. Moreover, we shortly characterize the tumor microenvironment and summarize how various metabolic factors within the tumor niche modulate antitumor capacity of NK cells. Moreover, we discuss the potential strategies and implications for the novel antitumor therapies augmenting NK cells functions.

**Abstract:**

NK cells have unique capabilities of recognition and destruction of tumor cells, without the requirement for prior immunization of the host. Maintaining tolerance to healthy cells makes them an attractive therapeutic tool for almost all types of cancer. Unfortunately, metabolic changes associated with malignant transformation and tumor progression lead to immunosuppression within the tumor microenvironment, which in turn limits the efficacy of various immunotherapies. In this review, we provide a brief description of the metabolic changes characteristic for the tumor microenvironment. Both tumor and tumor-associated cells produce and secrete factors that directly or indirectly prevent NK cell cytotoxicity. Here, we depict the molecular mechanisms responsible for the inhibition of immune effector cells by metabolic factors. Finally, we summarize the strategies to enhance NK cell function for the treatment of tumors.

## 1. Introduction

Natural killer cells (NK cells) have unique capabilities of tumor cells’ recognition and killing in a tightly regulated process shaped by a complex balance between inhibitory and activating signals [1]. This special competence of NK cells is gained in the course of development, maturation, and education in the bone marrow (BM) and secondary lymphoid organs, including lymph nodes [2]. NK cells found in the circulation are primarily divided into two subtypes: CD3^−^CD56^dim^CD16^+^ and CD3^−^CD56^bright^CD16^−^ cells [3] and they represent around 10% (5–20%) of circulating lymphocytes in humans [4]. CD56^bright^ NK cells have a higher capacity for cytokine production; however, they have relatively low cytotoxicity in comparison to CD56^dim^ cells [5].

NK cells develop from CD34^+^ hematopoietic progenitor cells (HPCs) residing in the BM, through a common lymphoid progenitor (CLP) to NK cells precursors (NKP), which are defined by expression of the IL-2/IL-15 receptor β chain (CD122), thus have the capacity for robust differentiation in response to IL-15 [6,7]. From the NKP stage, NK cells mature and begin to express molecules, such as NK1.1, NKp46, CD94 [8], and LFA-1 [9]. NK cells’ development, differentiation, functional maturation and survival is crucially dependent on BM stromal cells’ cytokines-induced signaling from the joint γ-chain cytokine receptors (IL-2R, IL-7R, IL-15R) (5) and subsequent activation of signal transducer and activator of transcription 5A/B (STAT5A/B) [10]. The mice with genetic defects in the IL-15/IL-15R system are characterized by the deficiency of NK cells [11], while IL-2–deficient mice have impaired NK cell response [12]. In the next stages of NK cells maturation, functional receptors CD161, CD56, NKG2D, and CD16 are expressed [13], while at the final stages of NK cells development CD56 is downregulated. NK cells are educated through the engagement of their inhibitory killer cell immunoglobulin-like receptors (KIRs) with various MHC class I molecules, resulting in the generation of functional NK cells [14]. Developing NK cells that interact with self-ligands through activating receptors become self-tolerant. After leaving the bone marrow, NK cells need to be activated comprehensively in secondary lymphoid organs to fulfil their antitumor role dependent on the release of cytokines and lytic granules. Naive NK cells acquire effector functions after a priming step—an interaction with dendritic cells (DCs) in draining lymph nodes, which results in their mutual regulation [15]. Trans-presentation of IL-15 by IL-15Rα on DCs stimulates the cytotoxic activity of NK cells and their ability to produce interferon-γ (IFN-γ) [16]. On the other side, NK cells activated by tumor cells modulate the adaptive immune response by inducing DCs’ maturation and activation. By killing tumor cells and releasing tumor antigens, NK cells stimulate tumor antigens cross-presentation by DCs in MHC class I context [17]. They also prime DCs to produce IL-12, thereby regulating activity and differentiation of functional T helper 1 cells (Th1), which in turn produce IL-21 involved in reciprocal stimulation of NK cells. NK cells can also directly influence T cells activity. CXCR3-dependent migration of NK cells to the lymph node stimulates, IFN-γ production, which successively promotes Th1 polarization [18]. Previous reports have demonstrated that IL-2, IL-12, IL-15, IL-18, and IL-21 play a significant role in NK cells activation and proliferation [19] via the stimulation of JAK/STAT signaling. STAT1 is a crucial regulator of IFN-γ production [20] and is also involved in cell junction formation at the NK cell-lytic synapse [21]. On the other hand, STAT3 has been described as a negative regulator of the NK cells cytotoxicity. The absence of STAT3 correlates with increased levels of perforin, granzyme B, as well as with the higher expression of activating receptor DNAM-1 [22]. STAT5 has been reported to be a master regulator of human and mouse NK cells’ activity. STAT5 inhibition in NK cells has been associated with tumor progression in vivo [10,23]. Specifically, IL-2 upon binding to its receptor [24], enhances NK cell response toward cancer cells, through the activation of the JAK-STAT5 signaling pathway. Likewise, NK cells stimulated by IL-15 rapidly increase the granzymes and perforin production, which is strictly regulated by the STAT5A/B activation [23,25] and PI3K-AKT-mTOR pathway [26,27]. It is worth noting that a combination of cytokines can induce synergistic effects on NK cell’s effector functions. It has been reported that IL-12 increases IFN-γ production in IL-15 stimulated NK cells [19,25] and together with IL-2 and IL-15 induces activation of STAT4 and STAT5 transcription factors. Also, direct binding of STAT4 to the perforin promoter has been described in IL-12 activated NK cells [28], IL-21 synergizes with IL-2 to augment the expression of NKG2A, CD25, CD86, CD69, and production of perforin and granzyme B [29].

## 2. Biological Aspects of NK Cell Cytotoxicity

### 2.1. NK Cells’ Metabolism

NK cells utilize glucose to fuel the biosynthesis of amino acids and fatty acids. Resting NK cells are characterized by relatively low rates of glycolysis and oxidative phosphorylation (OXPHOS), which then increases following the stimulation with IL-2, IL-12, or IL-15. Activated NK cells undergo metabolic reprogramming, leading to an increase in glucose uptake through glycolysis, which is supported by increased expression of nutrient receptors such as GLUT1, CD98, and CD71 [30]. As evidenced by the recent report, CD56^bright^ cells are more metabolically active than CD56^dim^ counterparts [31]. Furthermore, CD56^bright^ NK cells, due to their higher metabolic activity, can produce more IFN-γ during an immune response. The mammalian target of rapamycin complex 1 (mTORC1) is critical or NK cells development and maturation [32]. Although, the exact mechanism by which mTORC1 controls NK cells metabolism remains to be elusive. It has been reported that CD56^bright^ NK cells more robustly upregulate the activity of mTORC1 and increase glucose uptake and glycolysis upon cytokine stimulation [31]. Recently, it has been shown that cytokine-induced metabolic reprogramming of NK cells depends on the activity of two transcriptional factors: sterol regulatory element-binding protein (SREBP) and cMYC. SREBP controls elevated metabolism of glucose to cytosolic citrate in the cytokine-stimulated NK cells [33]. In the cytosol, citrate can be cleaved by ATP- citrate lyase (ACLY) to produce acetyl-CoA. SREBP activity, by controlling the expression of SLC25A1 (mitochondrial citrate transporter) and ACLY is essential for increased rates of glucose metabolism and [34]. Thus in NK cells, SREBP is fundamental for the modulation of glycolysis as well as OXPHOS via, regulation of the metabolic switch to citrate-malate-shuttle to fuel OXPHOS [34]. Therefore, SREBP inhibition may result in a reduction of NK cells growth, proliferation, and cytotoxicity against cancer cells by reduction of IFN-γ and granzyme B production [34]. mTOR has been described to have an essential role in promoting glycolytic metabolism in activated NK cells. mTORC1 activity is required for the initial increase in cMYC levels together with IL-2/IL-12 cytokine stimulation [34]. cMYC promotes OXPHOS and glycolysis by the upregulation of the glycolytic enzymes and glucose transporters [35,36]. Moreover, upregulation of cMYC in cytokine-stimulated NK cells heavily depends on the availability of the amino acids, especially glutamine [34,37]. Although glutamine is not an important nutrient for NK cells, cMYC protein expression has been reported to be sensitive to levels of glutamine. cMYC is also regulated by glycogen synthase kinase 3 (GSK-3), which has been shown to target cMYC for proteasomal degradation [36].

### 2.2. NK Cells Recruitment to the Tumor Site

Although the NK cell infiltrates have been identified in several cancers (e.g., melanomas [38,39] and breast cancers [40,41]), the precise mechanisms underlying trafficking of NK cells to the tumor microenvironment (TME) are yet to be discovered. Noteworthy, the infiltration of tumor sites by highly cytotoxic CD56^dim^ NK cells is often insufficient. Instead, cancer cells produce chemokines that stimulate the influx of less cytotoxic CD56^bright^ NK cells, whereas CD56^dim^ NK cells usually express receptors for chemokines produced at low levels within the tumor site [42,43]. All NK cells receptor chemokines are shown in Table 1, together with their respective ligands present in the tumor microenvironment or lymph node. Moreover, following cytokine stimulation NK cells modify the expression of chemokines and chemokine receptors. Therefore, the subsets composition and the tumor homing of the NK cell infiltrates can vary, depending on cytokines and chemokines present in the tumor microenvironment [44,45]. For example, IL-15 stimulates migration of CD56^bright^ NK cells to the tumor site by increasing the expression of CCR5, while inhibiting trafficking of CD56^dim^ counterparts by decreasing CXCR4 and CX3CR1 expression [46]. IL-2 upregulates chemokine receptors such as CCR1 and CX3CR1, and thus stimulates NK cells migration to the tumor site [47]. Also, it has been shown that IFN-γ stimulates tumor-infiltrating immune cells to release CXCL9-11, chemokines known to recruit CD56^bright^ NK cells [48]. Interestingly, a scarce NK cells infiltration has also been demonstrated in colorectal cancers despite high levels of NK cell-attracting chemokines within the tumor. Such findings suggest the presence of immune escape mechanisms that impair NK cell migration to the tumor site or decrease their viability within the tumor [49].

### 2.3. Formation of the Lytic NK-Cell Immunological Synapse

Following NK cells recruitment to the tumor site, the induction of NK cell effector functions requires direct NK cell contact with the tumor cell and formation of the lytic synapse. The formation of NK-cell lytic synapse ensures precise delivery and secretion of cytolytic effector molecules, leading to direct tumor cells death.

#### 2.3.1. Recognition Stage

**Figure 1:**
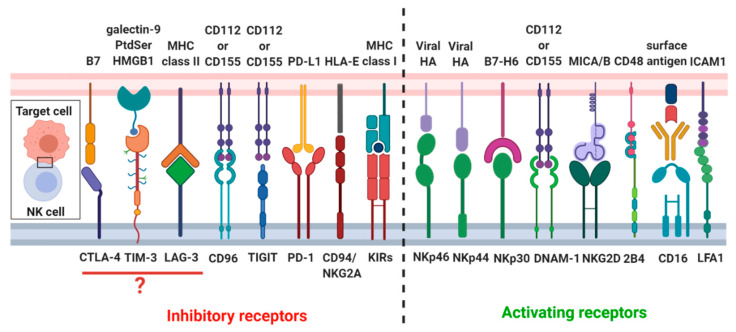
Overview of the NK cell receptors and their respective ligands. NK cells’ cytotoxicity is tightly regulated through the complex balance between inhibitory and activating signals originating from different NK cell receptors.

The formation of a mature and functional NK-cell lytic synapse is a complex process and can be divided into recognition, effector and termination stages. Recognition stage involves lytic synapse formation, which depends on the adherence of the NK cell to target cells. A variety of different molecules participate in lytic synapse formation, which includes NK cell receptors, intracellular signaling molecules, cytoskeletal elements and cellular organelles. A firm adhesion between two cells is established by interactions of high-affinity adhesion molecules, in particular, the NK cells express the intercellular adhesion molecules (ICAM) receptors: LFA1 (CD11a/CD18) [50,51,52,53]. After the initial contact of NK cells with the tumor cells, LFA-1 appears to initiate the process of synapse formation. Activation of NK cells cytotoxic activity is controlled by signals integrated from activating and inhibitory receptors (Figure 1) [54,55,56]. Among activating receptors, NKp30 and NKp46 are expressed on both resting and activated NK cells, whereas NKp44 is upregulated only upon NK cell activation [57,58]. Moreover, NK cells have the Fc receptor Fcγ RIIIa (CD16), which recognizes the Fc proportion of antibodies and triggers NK cell activation in a process termed antibody-dependent cell-mediated cytotoxicity [54]. Another important NK cell-activating receptors are DNAX accessory molecule-1 (DNAM-1) and NKG2D. Most of activating receptors of NK cells signal through the phosphorylation of the key Tyr residues in the cytosolic Tyr-based motifs (ITAMs) motifs. The phosphorylated ITAMs recruit the Tyr kinases ZAP-70 and Syk, which, in turn, phosphorylate transmembrane adaptor proteins, leading to recruitment of several signaling molecules, including the phosphoinositide 3-kinase (PI3K). On the other hand, inhibitory receptors such as KIRs, and C-type lectin inhibitory receptor CD94/NKG2A complex [54,55], inhibit NK cells activity. KIRs acts through immunoreceptor Tyr-based inhibitory motif (ITIM), which recruit the Tyr phosphatase SHP-1 or SHP-2. Several reports have also reported in NK cells the presence of non-canonical immune checkpoints receptors, such as PD-1 [59], cytotoxic T lymphocyte-associated antigen 4 (CTLA-4) [60], T-cell immunoglobulin and mucin-containing domain (TIM-3) [61], T cell immunoglobulin and ITIM domain (TIGIT) [62], CD96 [63], and lymphocyte activation gene 3 (LAG-3) [64]. PD-1 is highly expressed on activated T cells, but its presence was also reported in NK cells, mainly in CD56^dim^ NK cells subset [65]. PD-1 directly participates in NK cell exhaustion, limiting their cytotoxic activity and cytokines production [59]. Influence of CTLA-4 on the NK cells dysfunction remains very poorly understood. CTLA-4 might be involved in the inhibition of IFN-γ production by NK cells induced by DCs [60]. Additionally, it has been shown that IL-2-driven NK cells activation triggers CTLA-4 upregulation [60]. The role of TIM-3 in NK cells cytotoxicity is unclear, as both activating and inhibitory properties were described. On the one hand, TIM-3 blockade reduces NK cells cytotoxicity [66]; whereas, on the other side, high TIM-3 expression determines the subset of exhausted NK cells [67]. TIGIT and CD96 compete with activating receptor DNAM-1 for binding to CD112 and CD155 [68]; also both of them contain ITIM motifs in their cytoplasmic fragments, through which they deliver inhibitory signals to the target cell [69]. TIGIT is mainly upregulated on T cells, however higher expression of TIGIT has also been found on NK cells in intratumoral regions in colon cancer. Furthermore, blockade of TIGIT prevents NK cell exhaustion and promotes their cytotoxicity [70]. Moreover, LAG-3 expression has been observed only on the activated NK cells [71]. LAG-3 has been reported to be a NK cell negative regulator [69]; however, the direct impact of LAG-3 on NK cells function remains elusive. More study is needed, as currently only one group showed that inhibition of LAG-3 increases production of INF-γ, TNF-α, CCL3, and CCL4 [72].

#### 2.3.2. Effector Stage

At the effector stage, throughout synapse maturation, filamentous actin (F-actin) and adhesion receptors form a ring in the peripheral supramolecular activation cluster (pSMAC) zone, which is responsible for lytic granule secretion [53,73,74]. In the next stage, the lytic granules of NK cells dock onto the microtubule-organizing center (MTOC) in a process termed granule convergence. Then, the MTOC, along with the docked lytic granules, is polarized towards the NK-target cell synapses [52,75,76] by dynein–dynactin motor complex followed by docking of secretory lysosomes with the plasma membrane. The interaction between two cellular membranes, NK and target cell, is catalyzed by soluble N-ethylmaleimide-sensitive factor attachment protein receptors (SNAREs). Degranulation of NK cells is not only associated with pronounced cytoskeletal rearrangements but also is accompanied by mobilization of intracellular calcium and alterations of intracellular pH [77,78]. Stable NK-cell-target-cell conjugate enables secretion of preformed lytic granules, which are armed with perforin, serine proteases termed granzymes, and cathepsins [53,79]. An increase in pH and calcium concentrations triggers perforin release, its polymerization, as well as its subsequent binding and insertion into the target cells membrane, while granzymes induce target cells’ apoptosis [80]. Perforin is synthesized as premature, non-active form, which undergoes a multistep cleavage in acidic secretory lysosomes, leading to the formation of active perforin, which easily incorporates into target cell’s plasma membrane [81]. Granzymes A and B are the most abundant granzymes in NK cells. Similarly to perforin, in secretory lysosomes, granzymes are bind to the proteoglycans in the mechanism dependent on low pH. When the propeptides are removed, the inactive progranzymes become active proteases [82]. Human NK cells also produce high amounts of cathepsins, lysosomal peptidases involved in the regulation of effector stage of NK cells’ cytotoxicity by the processing of perforin, and granzymes.

#### 2.3.3. Termination Stage

The cleft that is formed at the lytic synapse between the NK cell and the target cell creates a protected pocket, which probably remains intact during a period of relative inactivity after the release of granules [83,84]. In this way, the concentration of the lytic effector molecules that are delivered to the target cell can increase while protecting neighboring cells from exposure to the cytotoxic molecules. At the termination stage, activating receptors previously recruited to the synapse, such as NKG2D and CD16, are downregulated [53]. Moreover, membrane flipping of the target cell-induced by perforin results in phosphatidylserine exposure on its external surface [80], which is recognized by ITIM-containing molecule CD300a [85] and provides a signal to terminate NK response. Once the NK cell has carried out its cytolytic function, it detaches from the target cell and restores its ability to kill another susceptible cell by generating new lytic granules and re-expressing activating receptors. The termination stage was shown to play a critical role in the process defined as ‘serial killing’. Termination stage is compulsory for NK cells to reinitiate a subsequent recognition and killing of a target cell [86].

### 2.4. NK Cells’ Cytokine Production

Although perforin/granzymes-mediated cytotoxicity is the most effective way of killing tumor cells, NK cells are now also known to promote slower receptor-mediated apoptosis and to produce cytokines. By secretion of IFN-γ and TNF-α, which activate resident inflammatory cells and recruit other cytotoxic immune cells, NK cells induce tumor cell death [87]. Production of IFN-γ by NK cells can be influenced by a cell to cell contact or by stimulation with IL-2, IL-12, or IL-15. It has been demonstrated that cytokine secretion by NK cells occurs independently from cytolytic granules release. In contrast to the release of cytolytic granules into the synaptic space, IFN-γ and TNF-α are delivered through multiple sites on the NK cells surface in a largely non-polarized mode [88]. The separation of these two effector pathways is an important mechanism allowing NK cells to simultaneously kill target cells and recruit other immune cells in antitumor response.

## 3. Characteristics of the Tumor Microenvironment

**Figure 2:**
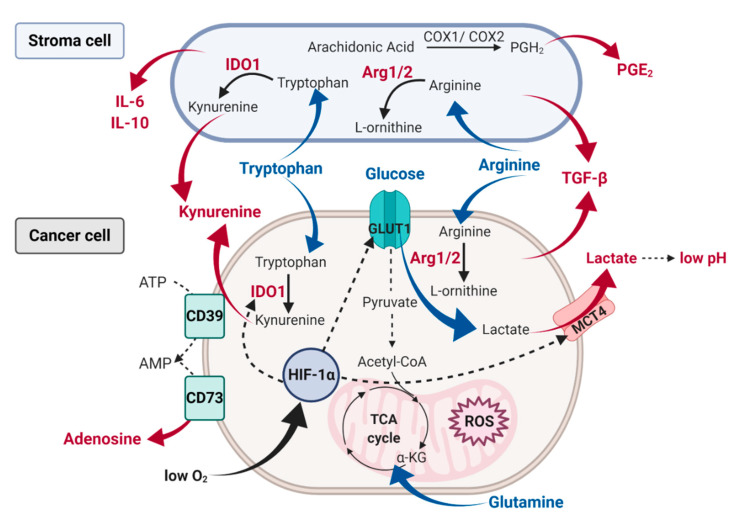
A schematic characteristic of the tumor microenvironment metabolites and other factors impacting NK cell effector function. Within the tumor microenvironment cancer cells consume large amounts of glucose and produce lactate and subsequent extracellular acidosis. Glycolytic conversion of glucose into pyruvate also stimulates the production of ROS. Tumor cells, as well as stromal cells, compete for nutrients such as glucose, glutamine, and amino acids. Thus, cancer cells together with stroma cells increase amino acids consumption and upregulate key amino acid metabolism enzymes, such as IDO and Arg1/2, leading to the accumulation of amino acids’ immunosuppressive metabolites, such as kynurenine. Tumor cells also generate extracellular adenosine through the ectonucleotidases CD39 and CD73. Moreover, high oxygen consumption by tumor cells can cause hypoxic conditions, which sustains HIF-1α, which in turn promotes glycolytic metabolism by upregulation of GLUT1 and lactate production by modulation of lactate transporters expression. Moreover, tumor and tumor-associated cells secrete factors, which prevent NK cell activation, such as TGF-β, IL-6, IL-10, and PGE_2_.

Tumor mass consists not only of malignant cells by also of stromal cells, infiltrating immune cells, blood vessels and a variety of extracellular factors, which constantly interact and shape tumor microenvironment (TME) during all stages of cancer development and progression [89]. Although the exact role of stromal cells in cancer development is context and cancer type-dependent in general, the overall function of stromal cells is beneficial for cancer cell survival and metastasis [90]. Within the tumor microenvironment, there are many types of stromal cells; however, the three major stromal cells populations are; cancer-associated fibroblasts (CAFs) [91], mesenchymal stem cells (MSCs) [92], and tumor associated macrophages (TAMs) [93]. Stromal cells together with cancer cells secret multiple factors to the extracellular space (Figure 2), which may have the immunosuppressive effect on the immune effector cells, thus limiting the efficacy of various immunotherapies, including NK cell-based therapeutic modalities. Nevertheless, it is worth mentioning that some of the systemic disorders, including obesity [94] and diabetes [95], may also influence tumor microenvironment. For example, during weight gain, adipocyte accumulates more lipids and undergo cellular hypertrophy and die [96]. This phenomenon results in increased concentration of numerous cytokines, such as IL-6, IFN-γ, and TNF-α, within the tumor microenvironment and subsequent cancer progression [95]. Therefore, in this part of the review, we summarize factors present in the tumor environment, which may shape tumor metabolism, development, and progression.

### 3.1. Tumor Hypoxia and Acidosis

In the majority of solid tumors, there are regions permanently or transiently subjected to the abnormally low level of oxygen. Environmental hypoxia results from inadequacies between the tumor microcirculation and the oxygen demands of the growing tumor mass. A central role in cellular adaptation to lower oxygen partial pressure is played by a hypoxia-inducible family of transcription factors (HIFs), comprising three members HIF-1, -2, and -3. Mechanistically, hypoxic conditions prevent HIF-1α from degradation by impairing its hydroxylation by prolyl hydroxylase-domain enzymes (PHDs) and subsequent polyubiquitination by von Hippel Lindau (VHL) tumor suppressor protein. Activation of HIF-1α leads to its accumulation, dimerization with HIF-β (aryl hydrocarbon receptor nuclear translocator) and subsequent nuclear translocation. In the nucleus, HIF-1 binds to the core DNA-binding sequence hypoxia response element (HRE) in the promoter regions and activates transcription of target genes. Among others, HIF-1 activation causes the upregulation of glucose transporters (GLUTs) and induces the transcription of glycolytic enzymes, particularly hexokinase, pyruvate dehydrogenase (PDH), and lactate dehydrogenase A (LDHA) [97]. This ultimately results in the ability of tumor cells to upregulate aerobic glycolysis, known as the Warburg effect. Glycolysis generates several intermediate products such as pyruvate, which can be transported to the mitochondria and used in the tricarboxylic acid (TCA) cycle. A large proportion of pyruvate is also converted in tumor cells to lactate by LDHA [98].

Moreover, a hypoxic TME is associated with a metabolic switch toward glycolysis and subsequent extracellular acidosis (pH < 6.8) and high (up to 40 mM) extracellular lactate (Figure 2). Under normal conditions, adenosine triphosphate (ATP) is generated from glucose slowly and efficiently by OXPHOS. However, tumor cells upregulate glucose uptake and lactate fermentation to sustain their increased metabolic demands [99]. Noteworthy, glycolysis produces ATP faster yet less efficiently than OXPHOS, therefore forcing tumor cells to consume much more glucose to maintain their high proliferative status [100]. Extremely low glucose, increased lactate and glycolytic intermediates concentrations are found in the tumor microenvironment (TME) in tumors of various origin [101]. When intracellular levels of lactate become too high, the proton-linked monocarboxylate transporters (MCT) pump lactate and protons outside the cell [102], leading to the further acidification of the tumor microenvironment. Likewise, when lactate is exported into the circulation, both local and distant tissues can utilize it as a fuel source. MCT are key players in this process: low-affinity lactate transporter MCT4 remove lactate from tumor cells and high-affinity transporter MCT1 ensures lactate uptake [103].

### 3.2. Oxidative Stress

Glycolytic conversion of glucose into pyruvate may stimulate reactive oxygen species (ROS) production in a mechanism dependent on mitochondrial membrane hyperpolarization [104,105]. Noteworthy, ROS regulate multiple cellular functions and can act as second messengers. For example, H_2_O_2_ modulates activation of signaling cascades of several growth factors and it can either induce cell death or proliferation depending on the dose [106]. Other active radicals, including nitric oxide (NO) belonging to reactive nitrogen species, owing to their high chemical activity, interact with multiple target molecules and play a pleiotropic role in cancerogenesis (summarized elsewhere [107]). Mitochondria-associated ROS are generated by electron transport chain, different isoforms of nicotinamide adenine dinucleotide phosphate (NADPH) oxidases (NOXs) and other enzymes including xanthine oxidase, lipoxygenase, or cyclooxygenase, as well as cytochrome P450 [108]. Under normal conditions, cells maintain a tight balance between ROS production and scavenging by cellular antioxidant enzymes [109]. Increase of glucose uptake by cancer cells is frequently observed under oxidative stress, a condition characterized by the imbalance between the generation of ROS and the antioxidant defense mechanisms [110]. Oxidative stress activates AMP-activated protein kinase (AMPK), which is meant to promote glucose-sparing oxidative metabolism, rather than aerobic glycolysis [111]. AMPK in turn activates pyruvate dehydrogenase complex (PDHc), a rate-limiting enzyme directly controlling pyruvate influx to the TCA cycle, which maintains TCA cycle and supports cancer metastasis [112]. Moreover, AMPK activation may occur under metabolic stress, such as glucose deprivation. AMPK increases cellular levels of NADPH, which subsequently neutralizes cellular ROS levels via NADPH-dependent synthesis of glutathione (GSH) and thus promotes tumor cell survival [113]. What is more, during hypoxia ROS levels increase and lead to the HIF-1α stabilization through PHDs inhibition [114,115,116]. In addition, HIF-1α can also be stabilized by phosphoinositide 3-kinase (PI3K) and p38 mitogen-activated protein kinase (MAPK p38) activated by hypoxia-derived ROS [117] and TGF-β [118]. Furthermore, TGF-β increases the production of ROS by impairing mitochondrial function and inducing NOXs activity. TGF-β also suppresses antioxidant systems, leading to oxidative imbalance [119].

### 3.3. Cytokines

Many cell types within tumor mass are described to contribute to the generation of an immunosuppressive tumor microenvironment by secretion of various cytokines. Accumulated in TME ROS participate in the regulation of downstream TGF-β signal transduction which involves SMADs, MAPKs, and NF-κB. In human cancer, TGF-β acts both as a tumor suppressor and as a promoter of tumor growth. The tumor suppressive effect includes inhibition of cell proliferation and induction of cancer cell apoptosis at the early stage of cancerogenesis [120]. On the other hand, tumor promoting effect includes induction of epithelial-to-mesenchymal transition (EMT), migration, and metastasis observed in aggressive and invasive tumors. In addition to cancer cells, a substantial source of TGF-β in the TME are TAMs [104,105,108] and neutrophils (TANs) [109], regulatory T cells (Tregs) [110], as well as myeloid-derived suppressor cells (MDSCs) [121,122]. These cells also secrete other cytokines, such as IL-6 [123] and IL-10 (Figure 2) [124]. Binding IL-6 and IL-10 to its receptors activate STAT3 tyrosine phosphorylation and subsequent transcription of target genes that support the tumorigenesis and maintain immunosuppression through MDSCs and TAMs [125]. Cancer cells can also produce IL-6 acting in an autocrine manner, in this way they do not depend on the paracrine release of IL-6 by stromal cells. Within the tumor microenvironment, various molecules support cancer cell growth and aggressive phenotype. However, it has been also shown that stressful and oncogenic stimuli, including cytotoxic agents and ionizing radiation, can induce senescence in cancer cells [126]. Unlike apoptotic cells, senescent cells are viable and secrete a wide array of immunomodulatory factors, including cytokines, growth factors and metalloproteinases, a phenomenon collectively termed as senescent associated secretory phenotype (SASP) [127]. Although NK cells are involved in the clearance of senescent cells [128], SASP can contribute to their inhibition in a mechanism involving metalloproteinase-mediated shedding of MICA and MICB, thus preventing activation of NKG2D receptors.

### 3.4. Amino Acid Deprivation

Close metabolic requirements of both immune and cancer cells for amino acids, like tryptophan (Trp), arginine (Arg), and glutamine (Gln) lead to the metabolic competition between them, resulting in amino acid depletion within the tumor niche [129]. Furthermore, under hypoxia glutamine consumption in cancer cells is elevated, which provides energy for cell survival. Glutamine promotes the TCA cycle and ATP production by being converted into α-ketoglutarate [130]. Importantly, glutaminolysis products activate mTORC1, hence promoting cell proliferation [131]. Amino acids can also help to counteract the negative influence of ROS within TME. For example, cancer cells depend on the glutamine for the synthesis of glutathione, which acts as an essential antioxidant in the cancer cells and maintains the redox homeostasis in the tumor niche [132].

On the contrary, glutamine and tryptophan deprivation leads to decreased mTOR activity resulting in inhibition of cancer cells growth. Furthermore, depletion of tryptophan, which is an essential amino acid for T-cell proliferation, depends on the indoleamine-2,3-dioxygenase (IDO) activity. IDO converts tryptophan into kynurenines (Kyn), which inhibit T and NK cells functions. IDO expression can be stimulated by various cytokines, including TNF-α, TGF-β, IL-6, and IFN-ɣ. In many cancer models, IL-6 was noted to modulate IDO expression through STAT3 phosphorylation [133]. What is more, IFN-ɣ can also promote IDO expression in DCs and MDSCs via STAT1 and STAT3 activity [134]. Nevertheless, tumoral expression of IDO can be inhibited by hypoxia and nitric oxide (NO) [135,136]. Although the low activity of IDO during hypoxia promotes the activation of immune cells [137], hypoxic conditions can also augment the secretion of IFN-ɣ, which in turn upregulates IDO mRNA expression [138]. Noteworthy, immunosuppressive properties of IDO can be further potentiated by PGE_2_, an arachidonic acid metabolite [139]. Other immunoregulatory enzymes within TME include arginase 1 (Arg1) and arginase 2 (Arg2), which catalyze the degradation of arginine. Both enzymes hydrolyze arginine into urea and L-ornithine, the main substrates for the production of polyamines required for cell cycle progression. Quantification of the murine tumor interstitial fluid metabolites revealed that arginine is one of the strongly depleted amino acids within the tumor microenvironment [140]. Noteworthy, there are several plausible explanations for this phenomenon. First of all, tumor-associated stromal cells seem to be the primary source of arginase within the tumor microenvironment [141,142]. They also express cationic amino acid transporter 2B (CAT2B), the transporter responsible for the rapid influx of Arg into the tumor-associated myeloid cells responsible for the depletion of extracellular arginine [143]. On the other hand, arginine may also be depleted by cancer cells [144,145,146], which overexpress Arg1 to sustain their rapid proliferation dependent on polyamine production. Moreover, some studies suggest that high Arg1 expression in TAMs is associated with enhanced tumor proliferation [147]. Arginine can also be metabolized by inducible nitric oxide synthase (NOS) to produce citrulline and NO, which are essential factors in tumor vascularization [143]. Reduced NO formation caused by arginine depletion may enhance ROS generation, which can further inhibit the activity of immune effector cells [148].

### 3.5. Alterations in the Key Enzymes of Lipid and Adenosine Metabolism

Arachidonic acid (AA) belongs to polyunsaturated fatty acids and is converted to various lipid-derived immune-mediators, including prostaglandins. The product of AA, prostaglandin H2 (PGH2) serves as the substrate for the isomerization to PGE_2_ [149]. The conversion is carried out by COX-1 and COX-2 enzymes. COX-1 is believed to be constitutively expressed in all tissues with the potential to induce an acute inflammatory response, whereas COX-2 is induced upon malignant transformation. Interestingly, H_2_O_2_ has been shown to induce COX-2 expression in a mechanism dependent on the inactivation of protein phosphatases activity and subsequent increased protein tyrosine kinases phosphorylation [150]. Prostaglandins and prostacyclins have a documented role in the modulation of the immune response. Secretion of PGE_2_ was confirmed in many cancer types and was associated with tumor progression and metastasis [151,152]. Within the tumor microenvironment, MDSC were also identified as the primary source of PGE_2_ and IDO1 (Figure 2) [153].

Adenosine (ADO), purine nucleoside, present at immunosuppressive concentrations within the solid tumor microenvironment, may also play a key role in immune evasion [154]. Two ectoenzymes- CD39 that hydrolyses ATP to AMP and CD73 that dephosphorylates AMP to adenosine constitute the main source of ADO in TME. Adenosine kinase is a cytosolic enzyme which controls ADO levels. Inhibition of adenosine kinase can effectively increase ADO extracellular concentrations. What is more, oxygen deprivation can also increase extracellular concentrations of ADO in tumor niche, since HIF-1 activation is responsible for the increased expression of CD73 [155,156].

## 4. How Tumor Microenvironment Factors Inhibit NK Cells

As previously described, induction of the NK cells’ cytotoxic activity involves several distinct stages, starting from the initiation of contact with a target cell to the directed delivery of lytic granules to the target cell. In this part of the review, we discuss how the tumor microenvironment could alter the balance between activating and inhibitory signals of NK cells, and also how it could shape NK cells’ priming and metabolism, which are essential to display full effector functions. We also review the effect of the TME factors on NK cells’ migration to the tumor site, degranulation, and expression of lytic granules’ enzymes.

### 4.1. NK Cells’ Metabolism

**Figure 3:**
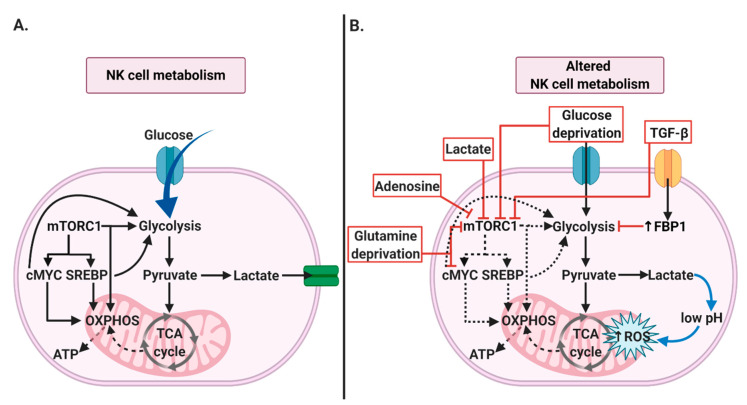
NK cell metabolism. (**A**) Key regulators of NK cells metabolism. Activated NK cells are characterized by increased glucose uptake and OXPHOS. mTORC1 is the key factor, controlling NK cell metabolism by upregulation of NK cells’ glycolysis and OXPHOS. mTORC1 is also involved in activation of cMYC and SREBP, which may further modulate glycolysis and OXPHOS. (**B**) Mechanisms disrupting NK cell metabolism in cancer. Many factors within the tumor microenvironment, can directly impact rates of glycolysis and OXPHOS by interfering with mTORC1, cMYC, or FBP1 activity. Moreover, mitochondrial dysfunction through ROS accumulation can be induced by intracellular lactate accumulation.

Activated NK cells upregulate glycolysis and OXPHOS in order to facilitate their cytotoxic function [157]. The primary regulators of the NK cell’ metabolism are mTOR, cMYC and SREBP (Figure 3A), which activity strongly depends on the nutrient availability within the tumor niche. Accelerated glucose metabolism in tumor cells is among others related to the reduced glucose availability, which represents a considerable obstacle for NK cells cytotoxic activity. When glucose levels are low, mTORC1 is inhibited, in turn leading to the repression of numerous anabolic processes. Impairment of mTORC1-maintained glycolysis in NK cells diminishes their cytotoxicity by inhibition of IFN-γ production and granzyme B expression [158,159]. Moreover, since cancer and immune cells compete for glucose, it seems that reduced glucose availability within the tumor microenvironment may represent one of the cancer strategies to suppress immune effector cells. For instance, Cascone et al. showed decreased T cell infiltration in tumors with high glycolytic rates [160,161]. However, whether glucose deprivation inhibits NK cells infiltration remains still an open question. Increased lactate uptake by NK cells can lead to their intracellular acidification, as evidenced by the ATP drop, indicating impaired NK cells’ energy potential [162]. Additionally, Harmon et al. have also shown that liver-resident NK cells treated with tumor conditioned medium (TCM) underwent apoptosis, which was associated with elevated lactate concentration within TCM [162]. Moreover, it has been demonstrated that lowering pH from 6.8 to 6.0 results in a significant decrease in NK cell activity [163]. It has been noticed that lactate accumulation and acidification of the extracellular environment cause dysfunction of NK cells by interfering with mTOR signaling, as shown in Figure 3B [159]. Also, it has been shown that lactate pretreatment inhibits the cytotoxic function of both human and mouse NK cells. Intracellular lactate decreases the intracellular pH and reduces ATP generation (Figure 3B) [164], which may cause ROS accumulation and mitochondrial stress with subsequent apoptosis of liver- resident NK cells [162]. It is worth noting that it has been shown that reduced lactate production by tumor cells results in slower tumor growth [165,166]. Likewise, a lactic acid concentration above 20 mM was shown to induce NK cells apoptosis, which might explain a smaller proportion of NK cells in tumors with a higher concentration of lactate, such as melanoma [162,167]. Moreover, under hypoxia, glutamine levels within TME are decreased due to elevated consumption by cancer cells. It has been noticed that glutamine withdrawal or SLC7S5 blockade results in the rapid loss of cMYC protein level and impaired NK cells cytotoxic response [34]. In the absence of cMYC, activated NK cells produced fewer IFN-γ and had reduced granzyme B expression [34]. Likewise, Cong et al. noticed that NK cells at later tumor stages were found in lower numbers and that loss of the antitumor effect of NK cells was closely associated with tumor progression [168]. Secondly, their transcriptome analysis of tumor-associated NK cells showed a strong upregulation of fructose- 1,6- bisphosphatase (FBP1) expression, a rate-limiting enzyme in gluconeogenesis (Figure 3B). Cong et al. showed that tumor-associated NK cells dysfunction and lower viability was strongly related to FBP1- mediated inhibition of NK cells glycolysis [168]. Additionally, short term hypoxia acts synergistically with IL-15 priming to induce the upregulation of genes involved in the glycolytic pathway [169]. However, the role of HIF-1α in NK cells effector functions and its effect on glycolytic activity remains unclear. In addition, the hypoxia-driven activity of CD73 impairs NK cells metabolism by generation of the highly immunosuppressive metabolite adenosine. ADO suppresses NK cells metabolic activity by inhibition of mitochondrial respiration and their proliferation through the mTOR pathway (Figure 3B) [170]. Likewise, besides cancer cells, NK cells themselves also can synthesize and secrete ADO within the tumor microenvironment. CD56^bright^ NK cells produce ADO via CD38 and CD203a, thus regulating other lymphocytes, while CD56^dim^ cells express lower levels of CD39 and CD73 [171]. Also, within the tumor microenvironment, amino acids deprivation may contribute to evasion of the anticancer immune response. For example, leucine depletion in tumor media was shown to inhibit mTORC1-dependent NK cell stimulation [172].

### 4.2. NK Cells Recruitment to the Tumor Site

According to literature data, the process of NK cells adhesion to endothelial cells in hypoxic conditions remains unchanged. However, in murine models, hypoxic mammary tumor cells support metastatic growth by secretion of cytokines and growth factors, which attract a specific subset of myeloid cells (CD11b+/Ly6Cmed/Ly6G+), as well as NK cells with reduced cytotoxicity [173]. Very recently, HIF-1α deficiency has been linked to reduced tumor growth, which was associated with non-productive angiogenesis and increased NK cell activity [174,175]. It has also been demonstrated that hypoxic conditions differently influences chemotactic responses of two functionally distinct human NK cell subsets in vitro. In tumors, CXCR4/CXCR7/CXCL12 pathway is involved in the complex scenario of tumor progression, including invasion, chemotaxis, and angiogenesis [174,176]. CXCL12 (a ligand for both CXCR4/CXCR7) promoter contains two HIF-1α binding sites. CXCL12, significantly upregulated in the tumor microenvironment, increases adhesion, migration, and homing of CXCR4-positive progenitor cells to ischemic tissues requiring regeneration and neovascularisation. Paroid et al. showed that hypoxia-induced CXCR4 and CXCR7 upregulation on CD56^bright^ NK cell population, thus induce migration to CXCL12 positive cells [177]. Therefore, the hypoxic environment may profoundly influence the nature of the NK cell infiltrate and its effects on immune-mediated responses within tumor tissues by promoting the accumulation of poorly cytotoxic CD56^bright^ NK cells [178]. It has also been shown that NK cells are a critical source of sVEGFR1 and thereby negatively regulate VEGF bioavailability in the tumor microenvironment. Deletion of HIF-1α in NK cells inhibits their ability to infiltrate tumor site and by increasing the VEGF availability and supporting non-functional tumor angiogenesis inhibits tumor growth [175].

**Figure 4:**
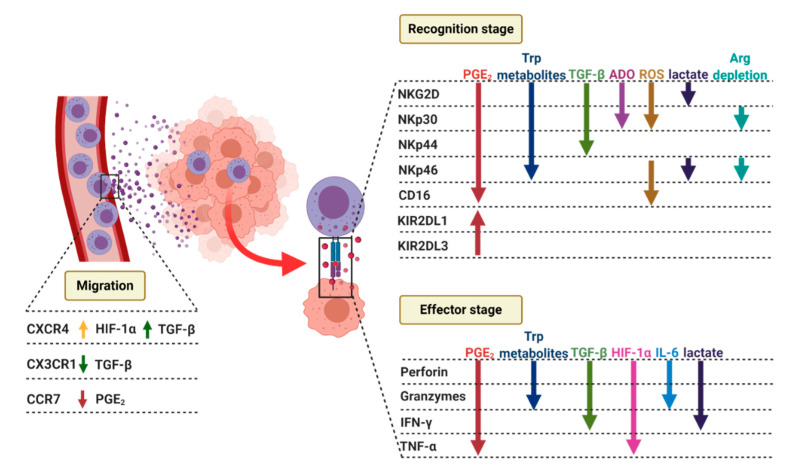
Tumor microenvironment shapes NK cells’ migration to the tumor site by upregulation of CXCR4 receptor through HIF-1α and TGF-β. It also downregulates CX3CR1 expression by TGF-β and CCR7 levels by PGE_2_, thus limiting their recruitment to the tumor sites. The formation of NK-cell lytic synapse can be divided into recognition, effector and termination stages. Within the tumor microenvironment factors such as PGE_2_, tryptophan metabolites, TGF-β, ADO, ROS, and lactate can downregulate NK cells activating receptors, including NKp46, NKp44, NKp30, and CD16 and inhibitory receptors, such as KIR2DL1 and KIR2DL3. On the other hand, during the effector stage, the same metabolites can decrease expression of the lytic granule molecules, such as perforin and granzymes. Also, they can influence NK cells’ ability for cytokine production, including IFN-γ and TNF-α.

Moreover, hypoxia also can limit the ability of NK cells to release chemokines involved in recruitment, differentiation, proliferation, and activation of APCs, Th1 lymphocytes, and NK cells; such as GM-CSF, CCL3, and CCL5 [177,179]. Likewise, TGF-β has been shown to modulate chemokine receptors repertoire, it downregulates the expression of CX3CR1 [180], but also increase expression of CXCR4 [181] (Figure 4). It has been noticed that extracellular messengers, such as ROS, could also guide NK cells to their destination. Previous studies have shown that H_2_O_2_ can recruit leukocytes to wounded sites [182] or oncogene-transformed cells [183]. Oxidative stress can also affect NK cell tumor infiltration. In gastric and esophageal cancer, H_2_O_2_ produced within tumor microenvironments inversely correlates with the infiltration of CD56^dim^ NK cells [184]. Several tumors, such as breast cancer [178] or non-small cell lung cancer [185], are characterized by the infiltration of CD56^bright^, poorly cytotoxic NK cells. NK cells decrease in number during lung cancer progression, and their intracellular ROS level is increased in the lung cancer microenvironment [168]. Moreover, ADO further potentiates the NK cell function inhibition. Among four adenosine receptors (A1, A2a, A2b, A3), A2a receptor (A2aR) is the most abundantly expressed on human NK cells and is responsible for the majority of immunosuppressive effects. Moreover, CD39 and CD73 were shown to interfere with the trafficking and cytotoxic activity of NK cells into solid tumor site through the heterologous desensitization of chemokine receptors [170]. NK cell homing to tumor tissue is also substantially changed by PGE_2_ [186,187]. It has been demonstrated that PGE_2_ inhibits migration of NK cells in response to SDF1a, MIP1a, ITAC, and CCL21 [151,188,189]. PGE_2_ also alters the NK cell chemokine receptor profile [187]. Within the tumor microenvironment PGE_2_ interferes with the production of CCL5 and CCL27 [187,188,189,190,191,192,193]. Since NK cells are responsible for dendritic cell recruitment to tumor tissue, PGE_2_-mediated inhibition of cytokine and chemokine production disrupts the NK-DC axis, causing a domino-like effect that impairs NK cell recruitment and other components of the anti-cancer immune response, leading to immune evasion and disease progression [187]. Trafficking of NK cells to inflamed tissues and tumor microenvironment was also shown to be affected by IDO metabolites [194,195,196,197].

### 4.3. NK Cells’ Lytic Synapse

#### 4.3.1. Recognition Stage

As described in detail before, tumor cells consume large amounts of glucose and produce lactate, which can accumulate in the TME and limit NK cells antitumor response. It has been observed that lactate-treated NK cells are characterized by decreased expression of activating receptor NKp46 as compared with the untreated cells, with no change in the level of NKp30, NKp44, and NKG2D [163]. In addition, the expression of NK cells surface molecules, such as CD18, CD54, and CD56, is reduced by acidic pH. While NK stimulation with IL-2 at neutral pH increased the expression of these molecules, in pH below 6.8 IL-2 lost its ability to modulate surface markers expression and did not affect the number of CD56^+^ NK cells [99]. Likewise, Crane et al. showed that LDH isoform 5 (LDH5) secreted by glioblastoma cells and detectable in sera from glioblastoma patients caused downregulation of NKG2D on NK cells via induction of NKG2D ligands on myeloid cells. Lower expression of NK cells activating NKG2D receptor decreased NK cells antitumor effect [198]. In another study, it has been shown that LDHA-deficient tumors are more responsive to anti-PD1 treatment. Similarly, LDHA blockade was shown to increase infiltration by NK cells characterized by increased IFN-γ production and higher granzyme B expression [199]. Moreover, targeting tumor acidosis has also been shown to increase the effectiveness of checkpoint inhibitors, including ant-PD-1 and anti-CTLA-4 [200]. Although PD-1 and CTLA-4 blockade is shown to increase mainly T cell activity, one can speculate that it presumably also enhances NK cells cytotoxicity [201]. Furthermore, within tumor microenvironment hypoxia is another factor which significantly reduces the expression of the activating receptors on NK cells. It has been demonstrated that in hypoxic environment NK cells lose their ability to upregulate the expression of NKG2D, NKp46, NKp30 and NKp44 in response to IL-2, IL-15, IL-12, and IL-21 [202]. Correspondingly, hypoxia has also been reported to increase the shedding [203] or downregulate the expression of an NKG2D ligand, the major histocompatibility complex (MHC) class I polypeptide-related sequence A (MICA), which correlates with a decreased susceptibility of tumor cells to NK cell-mediated cytotoxicity [204]. Interestingly, hypoxia does not significantly alter the surface density and the function of the FcγRIIIA receptor CD16, thus allowing NK cells to destroy target cells under hypoxic conditions via antibody-dependent cellular cytotoxicity [202]. Furthermore, downmodulation of NK cell-activating receptors, such as NKp46 and NKG2D, can also be triggered by ROS derived from phagocytic cells (Figure 4) [205]. However, this observation was limited only to the NK CD56^dim^ subset, while no changes were observed in CD56^bright^ cells, more resistant to redox stress. It has been shown that NKp46 is also downregulated in H_2_O_2_-treated CD56^dim^ cells [206]. It remains unresolved whether the down-modulation of activating NK receptors is related to the initiation of NK cell apoptosis by ROS or results from the direct effect of ROS on NK cell receptor expression. Interestingly, incubation of NK cells with PARP-1 inhibitor prevents ROS-induced NK cell apoptosis [207], which is accompanied by significant downregulation of NKp46 and CD16 with a modest decrease in NKp80 and DNAM-1 expression [208]. Downregulation of NKG2D, NKp30, NKp44 can also be a result of the production of high quantities of TGF-β in the tumor microenvironment (Figure 4). TGF-β also disrupts the NK cells ability to perform ADCC by CD16 downregulation [209]. Noteworthy, negative regulation of NKG2D by TGF-β- depends on the downregulation of DAP10 mRNA expression, which is an adaptor protein that stabilizes NKG2D on the cell surface and also transmits the phosphorylation events occurring upon NKG2D ligation [210]. ADO is also known to downregulate the expression of activating receptors NKG2D and NKp30, though their expression could be rescued by IL-2 priming [170]. Furthermore, recent studies have shown that Trp metabolites can decrease not only the expression of NKG2D but also NKp46, NKp44, NKp30, granzyme B, perforin, and CD69 in a kynurenine-dependent way (Figure 4). Interestingly, whereas Trp metabolites downregulate NKG2D, NKp46, TNF-α, and inhibit cytotoxicity in peripheral blood NK cells, they do not have any effect on decidual NK cells, suggesting a potential resistance of tissue-resident NK cells to kynurenine [211,212]. The abovementioned effects of Trp metabolites on NK cells are mediated via STAT1 and STAT3 pathways. First, kynurenine enters NK cells via the aryl hydrocarbon receptor (AhR) on the surfaces of the NK cells. Then, by disrupting STAT1 and STAT3 pathways with JNK inhibition being the critical event, it alters the NK cell phenotype [213]. It has also been shown that L-arginine deprivation within the tumor microenvironment reduces the expression of NKp30 and NKp46 and thus modulates functional properties of NK cells [214]. The cytotoxicity of NK cell was also shown to be inhibited by PGE_2_ [151,188,189,192,215,216,217,218,219,220,221,222,223,224]. The effect was dose-dependent and led to increased tumor burden in in vivo models [151,188,218,221,225,226,227]. The suppression of natural cytotoxicity was linked to PGE_2_-induced NKG2D downregulation [228]. Moreover, signaling from other receptors such as NKp30, NKp44, NKp46, CD16, as well as expression of Granzyme B and perforin is inhibited via PGE_2_ [192,215,216,222,224,228,229] (Figure 4). Additionally, PGE_2_ increases the expression of inhibitory receptors, such as KIR2DL1 and KIR2DL3 [216]. Since PGE_2_ suppresses NK cells within the tumor microenvironment, it also might inhibit DCs recruitment and subsequent NK-DC crosstalk.

#### 4.3.2. Effector Stage

A critical step in the establishment of the immunological synapse between tumor cells and NK cells is the translocation of the microtubule-organizing center and granules in NK cells toward the cell–cell contact region. In the target cells, actin cytoskeleton-dependent tethering of ICAM-1 and -2 (LFA-1 ligands), is required for proper integrin signaling in NK cells [230]. It has been shown that depolymerization of actin filaments in tumor cells inhibits the formation of immunological synapse mediated by LFA-1 and results in the impaired polarization of the granules. In hypoxia, remodeling of the actin cytoskeleton in the tumor cells can promote resistance to NK cell-mediated killing. Actin cytoskeleton remodeling in breast cancer cells has been linked to escape from NK-mediated cytotoxicity [231]. Specifically, hypoxia has been described to increase the expression and activity of actin-binding proteins and Rho GTPases (RhoA, RAc1, Cdc42). Subsequent stimulation of Rho GTPases-mediated actin and adhesion signaling pathways in tumor cells help them potentially to escape from the immune system control. It is also well documented that cancer cells can adapt to hypoxic stress through the activation of autophagy, a process responsible for the degradation of proteins and cytoplasmic organelles in well-characterized structures known as autophagosomes. It has also been reported that autophagy activation in breast cancer cells under hypoxia induces their resistance to NK-mediated killing.

Mechanistically, the resistance of hypoxic cancer cells to NK-mediated killing is related to selective degradation of NK-derived granzyme B in autophagosomes in target cells [232]. Intracellular granzyme B and perforin levels were also reported to be downregulated in NK cells in hypoxia (Figure 4); however, the exact mechanism of this phenomenon remains to be elucidated. Moreover, the decrease of granzyme B and perforin mRNA in NK cells can also be induced by lactate and low pH. Some reports suggest that the lactate may interfere with the secretory pathway of NK cytolytic machinery [163,233]. Diminished expression of granzymes A and B [234] and perforin can also be a consequence of long-term/chronic exposition of NK cells to cytokines, such as TGF-β or IL-6. IL-6 added to the human NK cells culture trigger the downregulation of perforin and granzyme B; however, a high level of IL-6 does not obscure the degranulation process performed by NK cells. The addition of tocilizumab, an IL-6 receptor blocker, rescues the expression of perforin and granzyme B and promotes cytotoxicity of NK cells [123].

### 4.4. NK Cells’ Cytokines and Chemokines Production

Hypoxia has been shown to change NK cells expression of proinflammatory cytokines, chemokines, and chemokine-receptors. In a comprehensive transcriptome analysis of hypoxic NK cells, Parodi et al. observed the downregulation of IFN-γ and several members of the TNF family, including TNF-α, LTA, LTB, TNFSF14, TNFSF10, and TNFSF11, which are involved in triggering tumor immunogenicity and decreasing tumor proliferation [177]. Recently, it has been shown that conditional deletion of HIF-1α in NK cells inhibits tumor growth, the phenomenon dependent on elevated expression of activation markers, effector molecules and an enriched NF-κB pathway in tumor-infiltrating NK cells. Accordingly, HIF-1α inhibitor increased human NK cell activity, and low HIF-1α expression was associated with high expression of IFN-ɣ in human tumor-infiltrating NK cells [174]. Moreover, lactate has been reported to impair the cytotoxic activity of PMA/Ionomycin-stimulated NK cells by inhibition of IFN-γ production, which likely promotes tumor immune evasion and growth. A possible explanation for the lower IFN-ɣ production may be the downregulation of nuclear factor of activated T cells (NFAT), key IFN-ɣ transcription factor [167,235]. On the other hand, the TGF-β family of cytokines orchestrate the multistep cascade of events resulting in the downregulation of the IFN-ɣ gene expression. The signaling of TGF-β is transmitted by TGF-β I and II transmembrane receptors and is associated with the phosphorylation cascade of serine and threonine kinases that mediate downstream SMAD2 and SMAD3 phosphorylation [236]. SMAD2/3 activation results in the direct downregulation of the IFN-ɣ gene as well as downregulation of the T-bet or E4BP4 transcription factors governing IFN-γ expression [237,238]. Another member of the TGF-β family, Activin- A, was also shown to activate SMAD2/3 pathway but by an alternative route —mainly by binding to ALK4 receptor. Similarly to TGF-β, activin-A can trigger the downregulation of transcription factor T-bet and subsequent IFN-ɣ. IL-10, at first, was shown to suppress NK cells expression of INF-ɣ and TNF-α in vitro [239]. However, in the light of recent findings, the influence of IL-10 in TME seems to be beneficial for NK cells, as it was shown to enhance the expression of genes engaged in NK cytotoxic and migratory activity [240]. Also, IL-2-induced IFN-ɣ, TNF-α, and GMCSF production were shown to be inhibited in NK cells by IDO [192,211,241]. Moreover, ADO inhibits TNF-α release from IL-2 stimulated NK cells. It has also been observed that in IL-15 stimulated NK cells. ADO increased the level of IFN-γ both in CD56^bright^ and CD56^dim^ NK cells [170]. Within the tumor microenvironment PGE_2_ also interferes with NK cells cytokines production. Disruption of IFN-ɣ, GM-CSF, and TNF-α synthesis was reported in multiple studies [187,188,189,190,191,192,193]. PGE_2_ in cAMP-dependent mechanism suppresses CCL5, CCL19, CXCL10, IL-12, IL-18 secretionand expression of ICAM-1 on dendritic cells, what was linked to decreased NK cell activation. Also, PGE_2_ inhibits NK cells ability to produce cytokines, such as IFN-γ [242] and TNF-α [216].

## 5. Strategies to Overcome the Inhibitory Effects of TME on NK Cell Functions

Although NK cells, representing the first line of defense against the tumor, are able to recognize and rapidly eliminate aberrant cells, TME constitutes one of the critical barriers to their activity (summarized in [236]). A complex interplay between tumor cells and surrounding TME cells occurring in the tumor microenvironment (TME) promotes the immune escape of tumor cells from NK cell-mediated surveillance, thus contributing to tumor progression. Suppression of NK cells within the tumor site is orchestrated by a variety of stromal, myeloid, and lymphoid cells, immunosuppressive cytokines, intratumoral nutrient availability, engagement of checkpoint molecules, and metabolic changes. Increased metabolic demands of tumor cells limit nutrient availability and expose tumor-infiltrating NK cells to various products of metabolic reactions that drive their functional exhaustion. Therefore, to overcome the NK cell limitations in their fight against tumor cells, several strategies have been recently introduced and explored at both preclinical and clinical levels [243]. The currently investigated main NK cell-based immunotherapeutics include the monoclonal antibodies (mAbs) neutralizing the immune checkpoint molecules, the adoptive transfer of ex vivo activated and expanded NK cells or chimeric antigen receptor (CAR)-modified NK cells (summarized elsewhere) [243]. Furthermore, understanding the metabolic changes and the immune suppression mechanisms present in TME has led to the development of new promising therapeutic agents and strategies. Here, we summarize the strategies targeting tumor cell metabolism designed specifically to support NK cells in hash TME by increasing nutrient availability to immune cells, decreasing acidity and hypoxia and reducing the production of immunosuppressive metabolites (Table 2).

## 6. Conclusions

In the immunosuppressive tumor microenvironment, NK cell priming, metabolism, and antitumor responses can be impaired by numerous factors. First of all, tumor cells compete with stromal cells and immune cells for the supply of glucose, glutamine and amino acids. In addition to reduced glucose availability, lactate accumulation and decreased pH within the TME also have a substantial impact on NK cell functionality. Therefore, targeting cancer metabolism represents a reasonable approach to improve the efficacy of NK cell-based therapies. Although most of the currently developed immunotherapies rely on the genetic modification of the cancer cells, strategies targeting cancer microenvironment offer novel and exciting possibilities. For example, CB-839, which is known to inhibit glutaminolysis without impairing NK cell cytotoxicity, could be utilized to augment immunotherapies. Additionally, NK cell cytotoxicity can be strengthened by targeting adenosine present in the tumor microenvironment, as few A2aR antagonists are currently tested. These compounds could potentially enhance NK cells maturation, activation and cytokine production. Similarly, NK cells’ cytotoxicity could be potentially strengthened by anti-CD73 antibodies. Furthermore, stimulation with different cytokine combinations can upregulate the expression of activating receptors and enhance NK cells lytic activity. Noteworthy, IL-2, IL-15, or IL-18 can also increase the expression of amino acid and glucose transporters. Therefore, it is necessary to continue exploring NK cell activity and to understand how it could be modified to resist the metabolically restrictive TME and preserve the effector functions, leading to the improvement in various immunotherapies.

## Figures and Tables

**Table 1:** Summary of chemokine receptors expressed by NK cells subpopulations and their respective ligands expressed by tumor or lymph nodes

Source of Chemokines	Chemokines	Chemokine Receptor	Chemokine Receptor Expression on the NK Cell Population	
			NK^bright^	NK^dim^
Tumor	CCL3, CCL5, CCL7, CCL9, CCL14-16, CCL23	CCR1	+	−
	CXCL1-3, CXCL5-8	CXCR2	−	++
	CXCL9-11	CXCR3	++	−
	CXCL12	CXCR4	+	++
	CXCL8	CXCR1	−	++
	CX3CL1	CX3CR1	−	++
	CCL3, CCL4	CCR5	++	+
Lymph nodes	CCL19, CCL21	CCR7	++	−

++ strong expression; + weak expression; − no expression.

**Table 2:** Strategies to overcome the inhibitory effects of TME.

TME Factor	Strategies	Examples of Clinical Trials (NCT: ClinicalTrials.gov Identifier)
Hypoxia	Priming of NK cells with IL-2 increases the expression of activating receptors and thus overcomes the inhibitory effects of hypoxia [244,245].	Natural Killer Cells Plus IL-2 Following Chemotherapy to Treat Advanced Melanoma or Kidney Cancer NCT00328861 Intraperitoneal Delivery of Adaptive Natural Killer (NK) Cells (FATE-NK100) With Intraperitoneal Interleukin-2 in Women with Recurrent Ovarian, Fallopian Tube, and Primary Peritoneal Cancer NCT03213964
	Modification of NK cells to increase ADCC potential and activity—high-affinity NK cells (haNK) expressing CD16 and IL-2 are resistant to acute hypoxia [246].	Phase 1 Study of haNK™ for Infusion in Subjects with Metastatic or Locally Advanced Solid Tumors NCT03027128
	Inhibition of HIF-1α (either by genetic modifications or small molecular HIF-1α inhibitor) enhances effector functions of activated NK cells (degranulation, production of IFN-γ and TNF-α [247].	None
Lactic acid/Low pH	Genetic blockade of LDHA (mice with LDHA deficiency) heightens infiltration of NK cells in the melanoma tumors. Infiltrated NK cells have an elevated production of IFN-γ and granzyme B [199]. Novel LDHA inhibitor reduces lactate production, thus decrease TME acidity [248].	None
	Bicarbonate monotherapy neutralises tumor acidity and increases effector cells infiltration [249,250].	Extended Use of Sodium Bicarbonate in Patients with Cancer NCT02531919
	Blocking the mitochondrial ROS accumulation to prevent NK cells mitochondria dysfunction and apoptosis [162].	None
Glucose depletion	FBP1 inhibition during tumor promotion, but not tumor progression, can restore NK cell function [168].	None
	GSK-3 inhibitors, CHIR99021, blocks proteasomal degradation of cMYC and thus promotes glucose consumption in NK cells [251]. CHIR99021 was shown to improve NK-cells function in ovarian cancer [251]. Moreover, other GSK-3 inhibitors, including LY-2090314 were shown to augment NK cells cytotoxicity in AML patients [252].	Phase 1 trials evaluating the application of NK-cells expanded ex-vivo and pre-treated with CHIR99021 in patients with AML (NCT03081780), ovarian cancer (NCT03213964) and other solid tumors (NCT03319459) Phase 2 trial of LY2090314 and Chemotherapy in Participants With Metastatic Pancreatic Cancer (NCT01632306)
ROS	Superoxide dismutase and other SOD-mimicking substances partially restore the NK-cell mediated killing of YAC-1 cells inhibited by superoxide [253].	None
	Histamine reverses granulocyte-induced inhibition of human NK-cell mediated killing of K562 cells [254]. Serotonin restores NK cell-mediated killing of K562 cells inhibited by mononuclear phagocytes [255]	Maintenance Therapy With Ceplene^®^ (Histamine) and IL-2 on Immune Response and MRD in Acute Myeloid Leukemia NCT01347996 -A Study of HDC/IL-2 Treatment in Chronic Myelomonocytic Leukemia (CMML) NCT03040401
	Catalase protects human NK cells from H_2_O_2_ induced apoptosis [206,253,256].	None
	Genetic inhibition of NOX2 (Nox2^−/−^ mice that lack the myeloid gp91^phox^ subunit of NOX2) or NOX2 inhibitor HDC reduces melanoma metastasis in a murine NK cell-dependent model of melanoma metastasis [257]. NOX2 inhibitors HDC and diphenylene iodonium chloride (DPI) play a protective role from monocyte-derived ROS-dependent NK cell apoptosis and mostly restore NK cell-mediated ADCC of primary CLL cells. NOX2 inhibitor HDC promotes degranulation of NK cells toward CMML cells in ADCC process and reverses CMML-induced NK cell apoptosis [208,256,257].	None
	ERK1/2 inhibitor PD98059 protects NK cell from H_2_O_2_-induced or monocyte-dependent apoptosis [207].	None
TGF-β	Chemical inhibitors- TGF-β receptor kinase inhibitor, galunisertib (LY2157299) improves the activity of NK cells in metastatic colon cancer mouse model [209].	A Study of Galunisertib on the Immune System in Participants with Cancer NCT02304419 ExIST Study of LY2157299 (Galunisertib) in Rectal Cancer NCT02688712
	anti-TGF-β antibodies- are shown to restore NK cells degranulation and cytokine release [258].	-Anti-TGF Monoclonal Antibody (GC1008) in Relapsed Malignant Pleural Mesothelioma NCT01112293 -Safety and Efficacy Study of GC1008 to Treat Renal Cell Carcinoma or Malignant Melanoma NCT00356460
	Genetic modification strategies- TGF-β dominant-negative receptor knockout receptor coupled to NK-activating domains (DAP12 or synNotch-RELA) enhance the cytotoxic activity of NK cells (particularly with DAP12 domain) [259]	None
Glutamine depletion	CB-839 It has been reported that glutaminolysis can be inhibited without reducing NK cell functional responses [34].	Study of the Glutaminase Inhibitor CB-839 in Solid Tumors NCT02071862
Tryptophan metabolites	IDO1 inhibition restores NKG2D expression on NK cells and promotes their proliferation [260,261]. IDO pathway inhibition enhances NK cell tumor infiltration and antitumor activity [262].	-Intraperitoneal Natural Killer Cells and INCB024360 for Recurrent Ovarian, Fallopian Tube, and Primary Peritoneal Cancer NCT02118285 -NLG802 Indoleamine 2,3-Dioxygenase (IDO) Inhibitor in Advanced Solid Tumors NCT03164603
	AHR antagonism increases cancer cell susceptibility to NK cell-mediated cytotoxicity and enhances NK cell-mediated ADCC [263].	-A First-in-Humans Dose Finding Study for an Aryl Hydrocarbon Receptor Inhibitor (AhRi) in Patients with Advanced Cancer NCT04069026 -IK-175 in Patients with Advanced or Metastatic Solid Tumors and Urothelial Carcinoma NCT04200963
	IL-18 treatment reversed IDO-mediated NK cell inhibition by upregulating NKG2D receptor [213].	None
Adenosine	A3R agonists: C1-IB-MECA increases activation and NK cells infiltration of B16-F10 melanoma. CF101 potentiation of NK cells’ activity [264,265,266].	None
	A2aR antagonists: SCH58261- enhances NK cells maturation, cytokine production, cytotoxic function against tumor cell lines, increases expression of granzyme B and reduces metastasis in a perforin-dependent manner. Increases NK cells infiltration of BRAF^V600E^-mutant melanoma. Promotes mouse NK cells proliferation and differentiation of human CD56^bright^ into CD56^dim^ mature NK cells. ZM241385- restores the cytotoxic function of IL-2 activated NK cells and cytokines production [267,268,269,270,271,272,273].	-A Study to Evaluate Immunotherapy Combinations in Participants with Gastrointestinal Malignancies NCT03720678 -A Study to Evaluate the Safety and Tolerability of Immunotherapy Combinations in Participants with Advanced Malignancies NCT03629756 - A Study to Evaluate Safety/Tolerability of Immunotherapy Combinations in Participants with Triple-Negative Breast Cancer or Gynecologic Malignancies NCT03719326
	CD73 inhibitor: APCP- reduces metastasis trough decreased A2aR-mediated suppression of NK cell-mediated cytotoxicity. Improves lytic activity of NK cells [270,273].	A Study of the CD73 Inhibitor LY3475070 Alone or in Combination with Pembrolizumab in Participants with Advanced Cancer NCT04148937
	anti-mouse CD73 antibody: TY/23- enhances anti-metastatic activity derived by NK cells [272]. Anti-human CD73 antibody increases the cytotoxicity of NK cells against ovarian cancer cell lines overexpressing CD73 [274].	A Study of AK119 (Anti-CD73) in Combination with AK104 (PD-1/CTLA-4) in Subjects with Advanced Solid Tumors -Study of GS-1423 (Anti-CD73-TGFβ-Trap Bifunctional Antibody) in Participants with Advanced Solid Tumors NCT03954704
	Anti-human CD39 antibody increases the cytotoxicity of NK cells against ovarian cancer cell lines overexpressing CD39 [274].	- Study of SRF617 (anti-CD39 antibody) in Patients with Advanced Solid Tumors NCT04336098 - TTX-030 (anti-CD39 antibody) Single Agent and in Combination With Immunotherapy or Chemotherapy for Patients With Advanced Cancers NCT03884556
	CD39 inhibitors: Polyoxometalate-1 (POM-1)- reverses Treg-mediated suppression of NK cells cytotoxicity and enhances their anti-metastatic activity. ARL67156- enhances the lytic activity of polyclonal NK cells [270,275,276].	None
Arginine	MDSCs upregulate arginase and catabolise arginine to NO. It has been found that NO impairs NK cell antibody-dependent cellular cytotoxicity and that the inhibition of iNOS can rescue this function [277].	
	Inhibition of the arginase activity by CB-1158 reduces tumor growth and increases tumor-infiltrating NK cells in vitro and in vivo [278]. OATD-02, another arginase inhibitor, has been shown to delay cancer progression [279,280].	Arginase Inhibitor INCB001158 as a Single Agent and in Combination with Immune Checkpoint Therapy in Patients with Advanced/Metastatic Solid Tumors NCT02903914
Arachidonic acid metabolites	Selective COX-2 inhibitors increase cancer cell sensitivity to NK cell-mediated lysis [281].	-Perioperative Administration of COX 2 Inhibitors and Beta Blockers to Women Undergoing Breast Cancer Surgery NCT00502684 -Perioperative Intervention to Reduce Metastatic Processes in Pancreatic Cancer Patients Undergoing Curative Surgery (BC-PC) NCT03838029
	EP2 antagonists restore tumor NK cell-mediated lysis [216]. EP4 antagonists restore NK cell antitumor activity cytokine production and migratory potential. Also, they decrease MHC I expression on cancer cells rendering them more sensitive to NK cell-mediated cytotoxicity [151,226].	Phase 1a/1b Study of TPST-1495 (EP2/EP4 antagonist) Alone and With Pembrolizumab in Subjects with Solid Tumors
